# No-scanning 3D measurement method using ultrafast dimensional conversion with a chirped optical frequency comb

**DOI:** 10.1038/s41598-017-03953-w

**Published:** 2017-06-16

**Authors:** Takashi Kato, Megumi Uchida, Kaoru Minoshima

**Affiliations:** 1 The Univ. of Electro-Communications (UEC), 1-5-1 Chofugaoka, Chofu, Tokyo, Japan; 2 JST, ERATO MINOSHIMA Intelligent Optical Synthesizer (IOS) Project, 1-5-1 Chofugaoka, Chofu, Tokyo, Japan

## Abstract

A simultaneously high-precision, wide-range, and ultrafast time-resolution one-shot 3D shape measurement method is presented. Simultaneous times of flight from multiple positions to a target encoded in a chirped optical frequency comb can be obtained from spectral interferometry. We experimentally demonstrate a one-shot imaging profile measurement of a known step height of 480 µm with µm-level accuracy. We further demonstrate the extension of the dynamic range by measuring in one shot a large step height of 3 m while maintaining high accuracy using the accurate pulse-to-pulse separation of the optical frequency comb. The proposed method with its large dynamic range and measurement versatility can be applied to a broad range of applications, including microscopic structures, objects with large size or aspect ratio, and ultrafast time-resolved imaging. This study provides a powerful and versatile tool for 3D measurement, where various ranges of measurement performances can be tailored to demand.

## Introduction

3D measurement techniques are widely required in fields, such as industrial measurement, sensing, robotics, and biomedical imaging. Several imaging methods have been developed, including laser speckle pattern sampling^1^, LIDAR^2, 3^, multiple-baseline stereos^4^, and optical coherence tomography^5^. Of these, distance imaging methods based on multi-point ranging^3^ are attractive because they can provide direct results without requiring complex analyses or model assumptions. The non-scanning method is particularly attractive because it can be applied to moving targets for rapid sensing and monitoring and to capture ultrafast phenomena. However, existing methods face problems in terms of performing one-shot 3D measurements with both high accuracy and large measurement ranges.

Previously, we proposed a novel principle for a one-shot 3D measurement method based on an ultrafast dimensional conversion between time, frequency, and space axis information encoded in chirped ultrashort pulses to capture dynamic 3D shapes^6^. This method was demonstrated using a highly chirped supercontinuum generated by an amplified Ti:Al_2_O_3_ laser and a femtosecond optical Kerr shutter to generate a real colour-coded 3D image in one pulse shot. This principle of simultaneous dimensional conversion using chirped pulses was applied to various area in several subsequent studies^7–9^. However, the previous study^6^ faced several technical challenges. First, the longitudinal dynamic range of the measurement, i.e. the ability to realise both the high resolution and a large range, was limited owing to the trade-off between the degree of the chirp and the spectral and temporal resolutions. Second, ultrafast time-of-flight measurements generally require pulse overlap between the reference and probe pulses via mechanical delay scanning. In addition, due to the laser technology limitations at the time, the light source was excessively bulky and unstable, and the image quality was poor due to the continuum generation by the bulk non-linear materials, all of which significantly limited the method’s accuracy as well as its practical applicability.

Recently, optical frequency combs (OFCs) have revolutionised frequency metrology^10, 11^ and provided tools for the precise control of light waves with extreme accuracy. This technology is useful in various application fields, including distance measurements with extreme accuracy and dynamic range^12–15^. Further, fibre-based OFC technology is providing mature solutions for various practical applications^16, 17^. However, its potential has not been completely explored in fields other than precision metrology.

In this study, we propose a new one-shot 3D measurement method using rapidly advancing OFC technology. This study will allow OFCs to be used in fields beyond precision metrology. Based on our previous chirp-imaging method’s principle^6^, pulse-to-pulse interferometry using OFCs^18^ significantly extends the longitudinal measurement range based on high-precision pulse-to-pulse separation and could possibly break the fundamental trade-off in measurements to achieve extreme dynamic ranges that include the simultaneous imaging of separate targets with m-order distances without losing high precision. Moreover, spectral interferometry provides a linear time-gating method to detect chirp information with a practical low-power laser such as fibre laser. By taking full advantage of the recent technological advancements in OFCs, high accuracy, dynamic-range, ultrafast time-resolution, and controllability can be simultaneously obtained in 3D measurement techniques.

## The Measurement Principle

**Figure 1:**
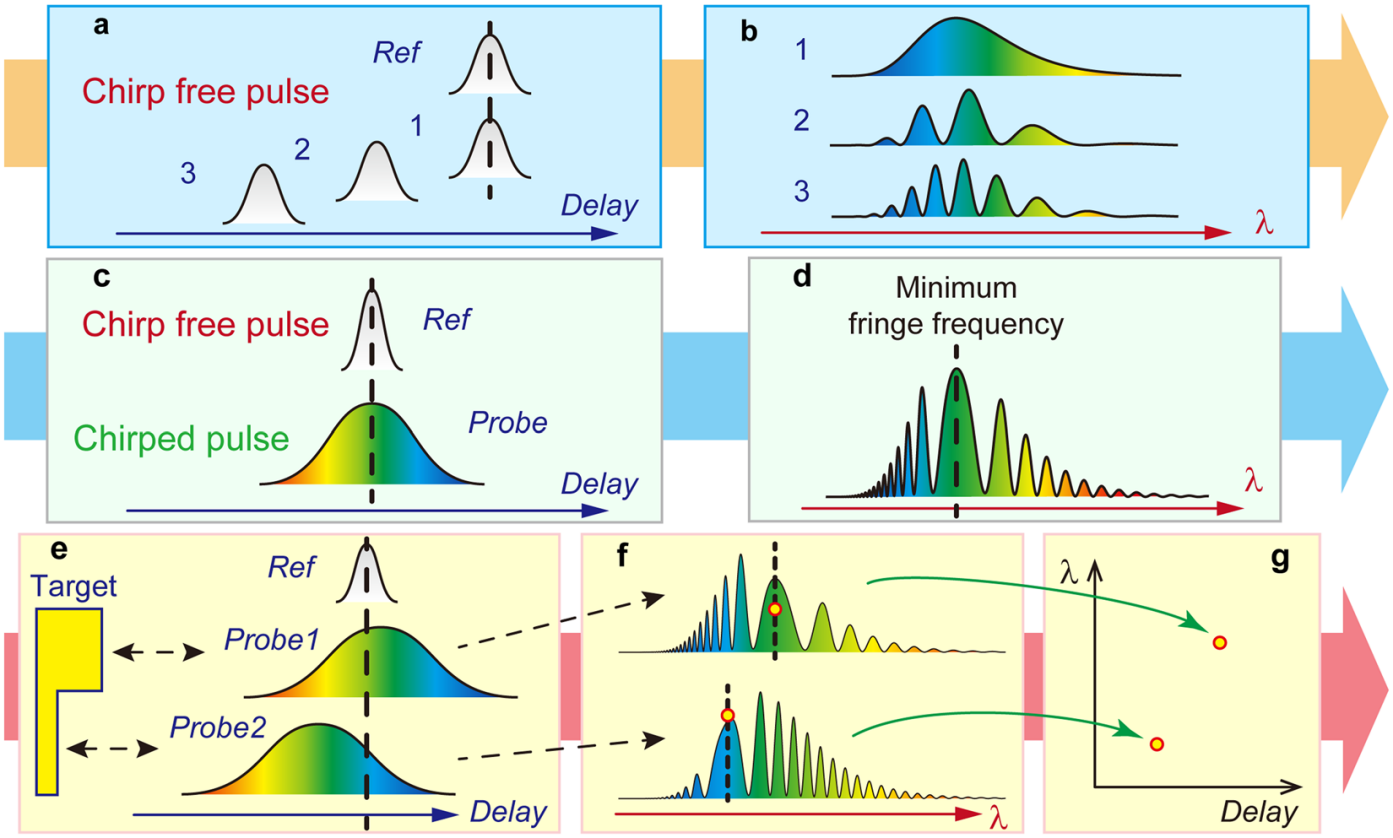
Measurement principle based on spectral interferometry with chirped pulses. Spectral interferometry with a chirp-free reference pulse and (**a**) chirp-free and (**c**–**g**) chirped probe pulses. (**a** and **c**) Space diagram of the pulses. (**b** and **d**) Schematics of interference fringe spectra. Minimum fringe frequency (MFF) indicates the region showing the broadest fringe. (**e**) Space diagram of reference and probe pulses reflected from a target. (**f**) Schematic of interference spectra. (**g**) Relation between wavelengths showing MFF and delay.

The proposed method is based on spectral interferometry^19–22^ between a chirped probe and chirp-free reference pulses (Fig. 1). In conventional spectral interferometry between the two chirp-free pulses, a uniform interference fringe spectrum is observed wherein the fineness of the fringe, i.e. the fringe frequency, is constant along the optical frequency (waveleng﻿th) and varies depending on the delay time between the two pulses (Fig. 1a,b).

Conversely, if one of the pulses (i.e. the probe pulse) is chirped, a non-uniform fringe spectrum is observed wherein the wavelength region showing the broadest fringe, i.e. the minimum fringe frequency (MFF), represents the wavelength component in the chirped pulse that has a temporal overlap with the reference pulse (Fig. 1c,d). In the shape measurement, the target shape is converted to the difference in the time delay of the reflected chirped probe pulses, resulting in a difference in the wavelength regions with MFFs (Fig. 1e,f). Therefore, measuring the wavelength showing the MFF can provide the delay time, i.e. the shape information (Fig. 1g). Finally, irradiating the expanded probe beam over the entire area of a target and capturing the spectral interference fringe pattern with a 2D spectrometer allows the 3D shape of the target to be measured with one shot of the pulse. Once the calibration curve is obtained by measuring the wavelength of the MFFs while changing the known delay time, the unknown distance can be measured without changing the delay.

In conventional pulse interferometry, the reference and probe pulses must be aligned using a mechanical stage so that they overlap within a pulse width. Using pulse-to-pulse interferometry with OFCs, precise and rapid adjustment of the delay can be achieved via frequency control prior to the initial setting, and the target can be measured at arbitrary positions without a mechanical stage^23^. Moreover, using a precise pulse-to-pulse separation significantly enhances the measurable range without losing longitudinal resolution, enabling the proposed method to simultaneously obtain large dynamic range and high precision.

In this way, the longitudinal measurement is based on the newly developed unconventional length measurement technique using chirped frequency comb, but, on the other hand, the transverse measurement is based on the conventional colour imaging technique whose performance is determined mainly by the optical system. Therefore, in the experimental demonstration, we performed the proof-of-principle of the proposed method by precise measurements of the step structures and comparison with length standards made of gauge blocks.

## Results

### Point distance measurements

As a first proof-of-principle experiment, the longitudinal distance to a point target was measured by detecting the change in the spectral interference fringe as the delay time changed.

**Figure 2:**
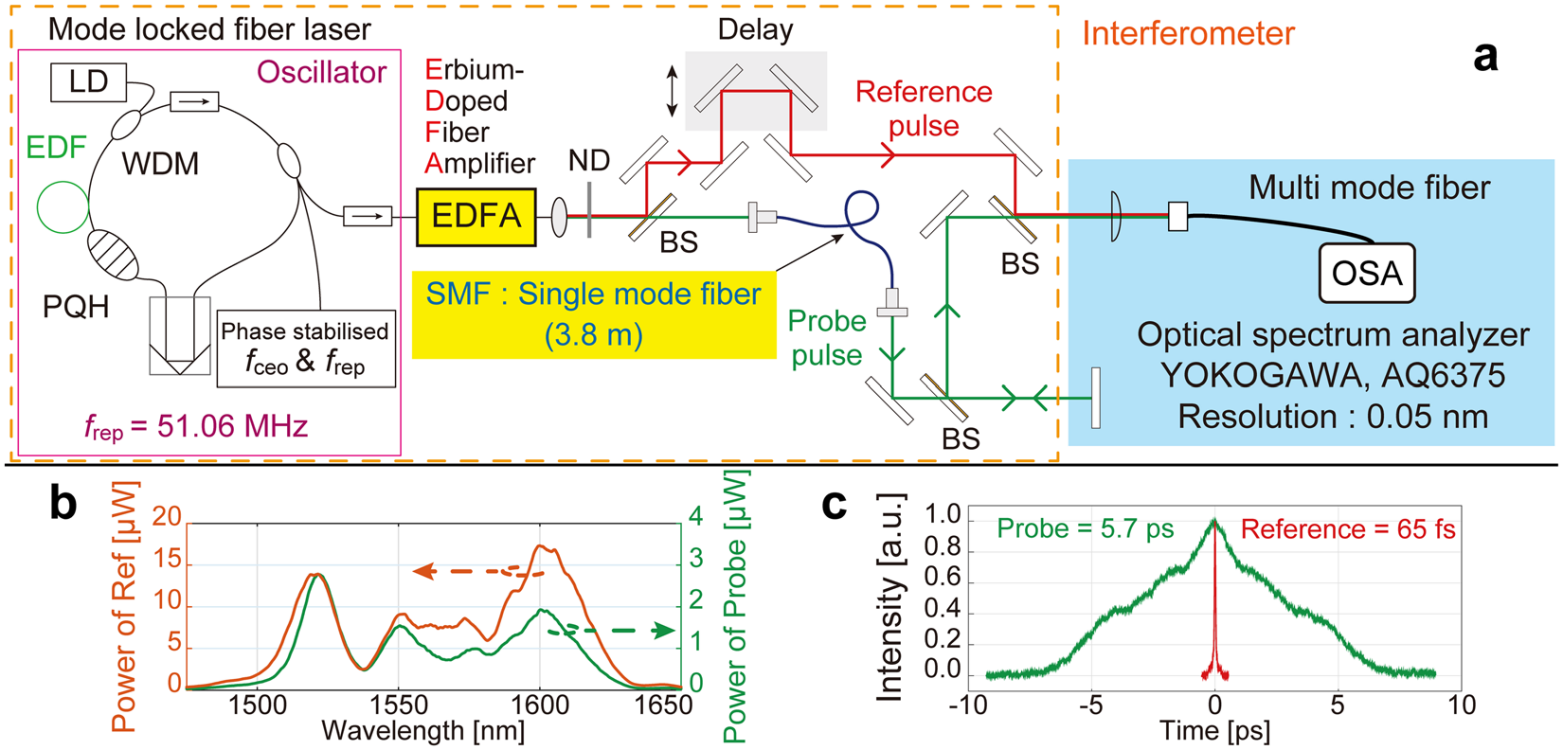
Schematic of the experimental setup. (**a**) Experimental setup for the position measurement. LD: laser diode, WDM: wavelength division multiplexing coupler, PQH: polarization controller, EDF: erbium-doped fibre, ND: neutral density filter, and BS: beam splitter with *f*_rep_: 51 MHz, central wavelength: 1.56 µm, and average power: 8 mW after EDFA. (**b**) The optical spectra and (**c**) autocorrelation traces for the reference and probe pulses.

Figure 2a shows the experimental setup. The light source was a home-made mode-locked Er-doped fibre laser with a phase stabilisation of the repetition frequency *f*_rep_ and the carrier-envelope-offset frequency *f*_ceo_ of the comb to the microwave frequency reference with an uncertainty of 10^−11^. It comprised an unbalanced Mach–Zehnder interferometer with an arm difference corresponding to the pulse-to-pulse separation of the comb. The reference pulse was chirp-free, and the probe pulse was highly chirped via the propagation of a single-mode fibre (length: 3.8 m) (Fig. 2b,c). The probe beam was irradiated onto a fixed plane mirror, and the interference fringe spectrum was detected using an optical spectrum analyser.

**Figure 3:**
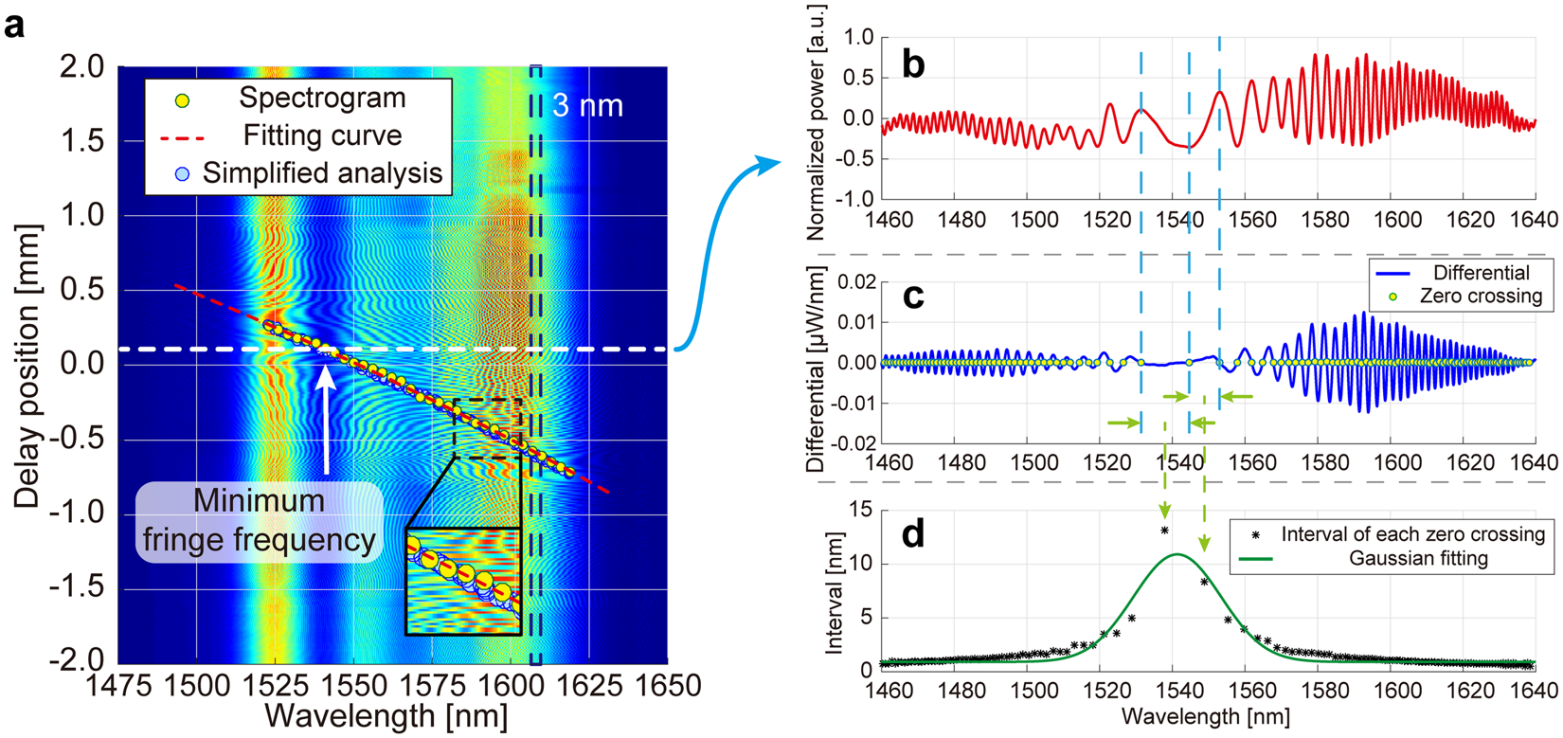
Results of the position measurement. (**a**) False colour plot of spectral interference fringes obtained by changing delay positions. A series of data points corresponding to minimum fringe frequencies (MFFs) are plotted. The 33 yellow circles labelled ‘Spectrogram’ were obtained via FFT, while the blue circles labelled ‘Simplified analysis’ were obtained via the differentiation of the spectrum (**b**–**d**). Details can be found in Methods section.

Figure 3a shows a false colour plot of a series of interferometric fringe spectra obtained when the delay was varied by moving the stage. The fringe patterns showing distinct fringe structures, i.e., the MFFs, clearly moved towards the shorter wavelength side with increasing delay. From the 2D plot, we obtained 33 MFFs using a short-term Fourier transform along the delay (plotted as the yellow points in Fig. 3a labelled ‘Spectrogram’; details can be found in the Methods Section). Consequently, the corresponding ‘wavelengths’ showing the MFFs display a monotonic behaviour with an approximately quadratic dependence on the delay, which reflects the chirp behaviour of the probe pulses. The residual of the quadratic fitting (‘Fitting curve’ in Fig. 3a) produces a standard deviation of 10 µm, which can be further reduced by optimizing the measurement system and the analysis.

In this way, the relation between the delay and the wavelength corresponding to the MFF can be obtained and used as a calibration curve for the measurements. In actual applications of one-shot 3D measurement, the delay is fixed at a certain position and the spectral interference fringe pattern is measured in one shot (white dashed line in Fig. 3a). Next, the fringe spectrum is analysed to obtain the MFF wavelength based on the differentiation of the spectrum (the ‘simplified analysis’ described in the Methods section). In Fig. 3a, the same data were analysed using this procedure (the blue points in Fig. 3a labelled ‘Simplified analysis’), and the results were compared with the previous analysis using FFT. The difference between the previous fitting results shows a standard deviation of 8.2 µm, which is of the same order as the data obtained by the ‘Spectrogram’, confirming the consistency of the two analyses. Therefore, we can measure the distance with an approximate uncertainty of 10 µm in one shot without moving the delay.

### Step height and profile measurement

**Figure 4:**
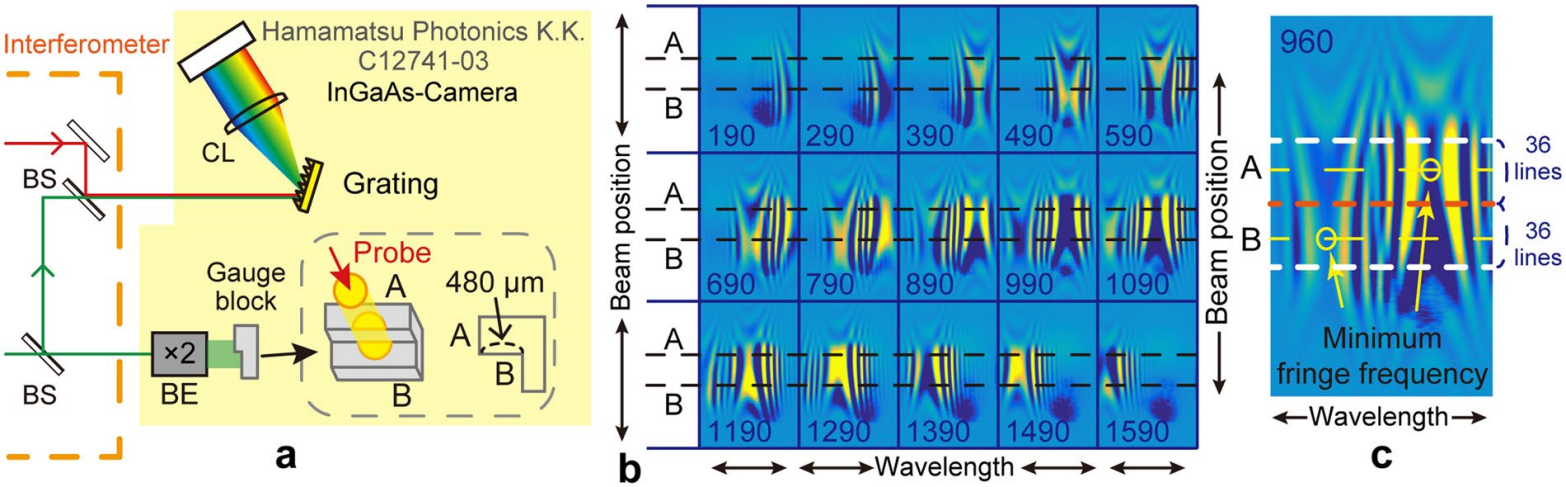
Schematic of the step measurement and captured images. (**a**) Experimental setup for the step measurement. Interferometer setup is shared with setup shown in Fig. 2a. BE: beam expander and CL: cylindrical lens. Grating: diffraction grating, 600 line/mm. (**b**) Captured spectral interferometric images by InGaAs camera at each delay position (number in each image, in µm unit). The images were processed so that the contrast was enhanced, and the horizontal and vertical directions of each image represent the wavelength and beam positions. Black dashed lines in each image are guide for eyes indicating area of steps A and B. (**c**) Zoomed image is at a delay position of 960 µm. To obtain longitudinal heights for steps A and B, 36 horizontal cross-sectional lines between upper and bottom white lines and central red dashed line were analysed. Horizontal yellow dashed lines indicate central area in the regions of steps A and B.

Next, we performed one-shot measurements of a step of known height comprising two gauge blocks and evaluated the absolute uncertainty of the measurement. In this case, the probe beam was expanded using a beam expander to a diameter of 4 mm and was subsequently irradiated onto the area containing the optically contacted two-gauge block step, resulting in a precisely 480-μm-high step (Fig. 4a). The spectrally resolved interference fringe pattern was captured via a camera, and the obtained 2D image with the wavelength and spatial (beam position) information in each direction enabled the measurement of the height between steps A and B in one shot.

**Figure 5:**
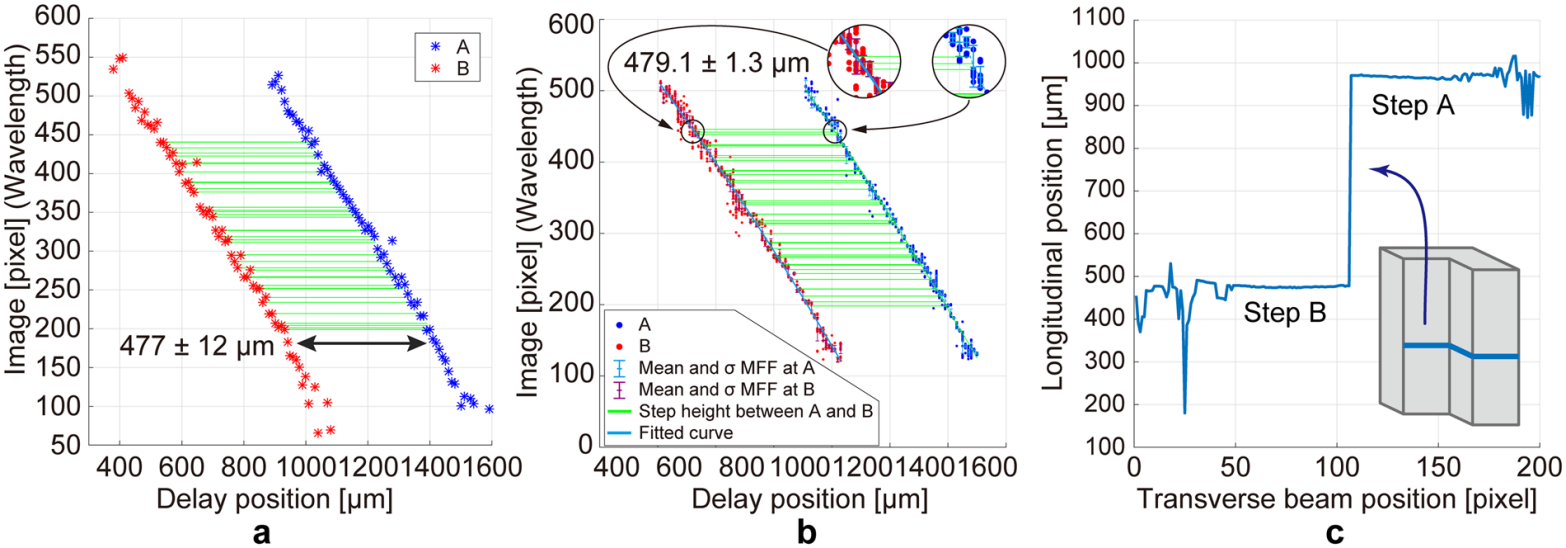
Results of the step measurement. (**a**) Series of MFFs corresponding to the central areas of steps A and B. (**b**) Series of MFFs for all 36 beam positions obtained by changing delay positions. Detailed explanations can be found in the text. (**c**) Profile for the step shape of gauge blocks obtained from one-shot imaging at a delay position of 960 µm (Fig. 4c). Overlapping regions between the areas A and B due to the diffraction of the edge are not shown and the edge position was determined based on the rough estimation of the irradiated beam position.

Figure 4b shows a series of captured images, where two fringe patterns corresponding to the two areas A and B were clearly visible and moved as the delay changed. Owing to the diffraction at the edge of the steps, the two fringe patterns had overlapping regions. From each image, we picked the two beam positions corresponding to the central areas of A and B and analysed the fringe spectra. As an example, the beam positions used for the analysis of the delay position 960 µm are shown in the zoomed view in Fig. 4c as two yellow dashed lines. In Fig. 5a, the red and blue points represent the series of obtained MFFs corresponding to steps A and B, respectively. The separation of the delay positions between the red and blue data points sharing the same wavelength were calculated using linear interpolation to find the counterparts. Only a part of the data from 190 to 450 pixels on the wavelength axis were used in the analysis (the 39 green lines in Fig. 5a) because the edge areas in the images corresponding to 190 pixels which is the width of the broadest fringe could not provide a central peak with high accuracy. Finally, the obtained step height was 477 ± 12 μm, which agrees well with the nominal value of 480 μm (Fig. 5a). The uncertainty was estimated from the standard deviation of the 39 data, which can be further reduced with an improved optical setup with less crosstalk in the captured images.

Next, we performed a similar analysis for all the transverse beam positions corresponding to a series of 36 horizontal lines in each image indicated in Fig. 4c. A series of MFFs were obtained for all 36 beam positions by changing the delay for the step areas A and B (the red and blue dots in Fig. 5b, respectively). The average of the wavelengths giving the MFFs in the transverse beam positions for each delay was obtained and a series of step heights was calculated using the same procedure discussed above (the 40 green lines in Fig. 5b). Finally, averaging all the obtained values, a step height of 479.1 ± 1.3 μm was obtained, which is in ideal agreement with the nominal value. Therefore, the proposed method can provide an accurate longitudinal position, i.e., distance, measurement with 1 µm-order uncertainty. This result suggests that by improving the optical and detection systems, achieving one-shot 3D measurement with 1 µm-order uncertainty is possible.

Finally, the line profile of the step built from the gauge blocks was obtained from one shot of the image at a fixed delay of 960 µm (Fig. 4c), as shown in Fig. 5c. In this analysis, we used a calibration curve for the relation between the wavelengths of the MFFs and the delay positions, i.e., the actual longitudinal positions, obtained from the quadratic fitting (the blue line in Fig. 5b). This calibration process with delay scanning is only necessary to do once before actual measurement of the target. In this way, we confirmed the precise measurement of the step profile using one-shot imaging.

### Large step height and profile measurements

Because the step height used in the previous section was small, parts of the same probe pulses that were reflected from the step areas A and B simultaneously overlapped with the same reference pulse. Instead, if a series of different pulses in a mode-locked pulse train were simultaneously used as the probe, it would be possible to measure much larger distances, e.g., at the metre scale, with the same µm-order accuracy because the m-order repetition distance of the OFC pulse trains can be precisely determined. This method uses two advantages of OFCs: high coherence and accurate *f*_rep_.

**Figure 6:**
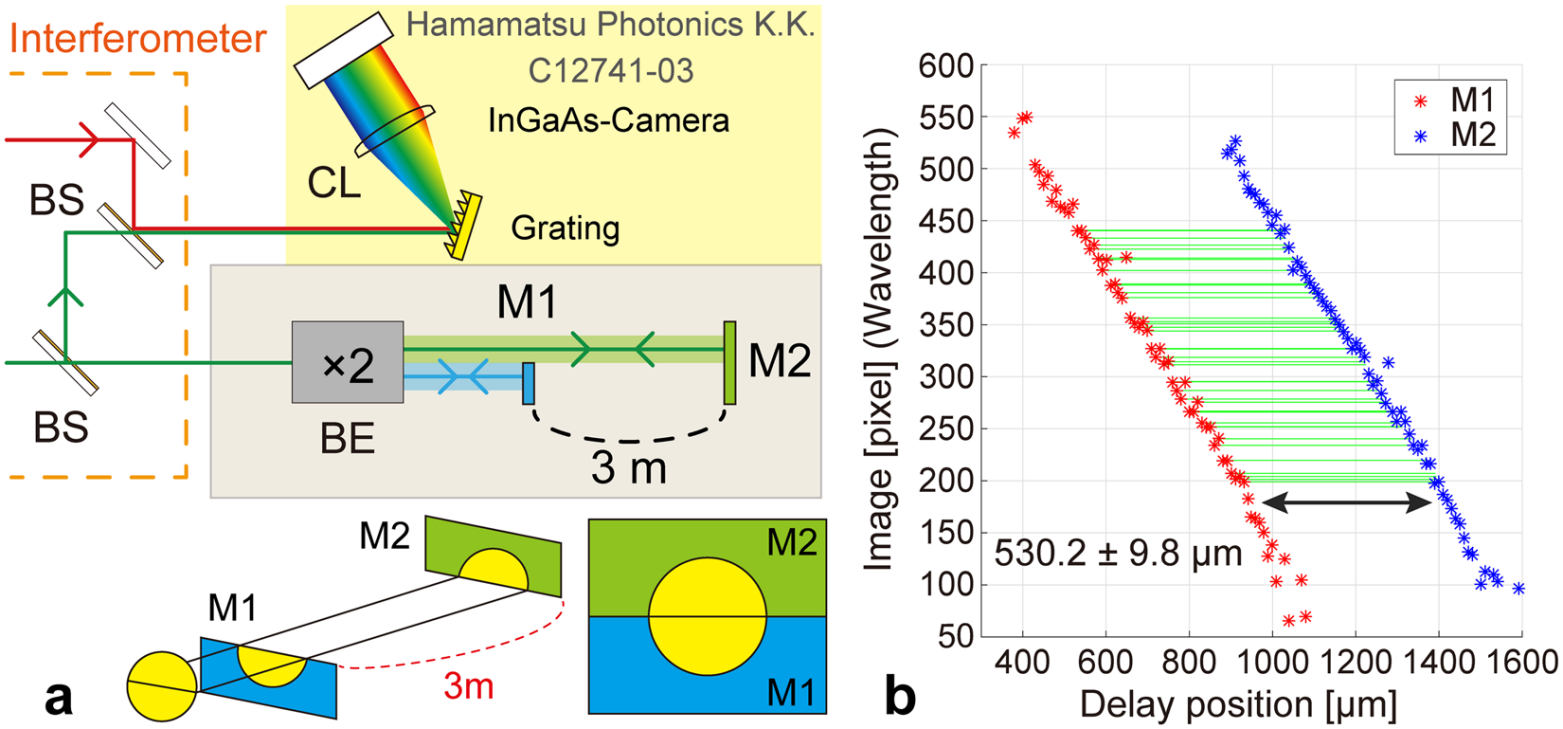
Schematic and results of the large step measurement. (**a**) Experimental setup for the large step measurement. The interferometer setup is same as that shown in Fig. 2a. (**b**) Series of minimum fringe frequencies obtained for the two m-sized separated mirrors.

To demonstrate this principle, we measured a large-size step (height: 3 m) with successive probe pulses. In this case, the probe beam was simultaneously irradiated onto two mirrors placed 3 m apart, called M1 and M2 (Fig. 6a), whose separation is nearly equal to the pulse-to-pulse separation. The two successive pulses in the pulse train reflected from M1 and M2 were allowed to simultaneously interfere with the same reference pulse, and the two spectral interference fringes were obtained by one-shot using a grating and subsequently captured via a camera in a manner similar to that described in the previous section.

Figure 6b shows the results for a series of wavelengths of the MFF at each mirror. Similar to the previous section, we obtained the distances between the red and blue points (the 38 green lines in Fig. 6b). The average of the obtained distances was 530.2 ± 9.8 µm. Using the precise value of the OFC *f*_rep_, 51.07262 MHz, which was determined with an uncertainty of 10^−11^, we measured the distance between the two mirrors at 2.9347108 m with an uncertainty of 9.8 µm. Here, the multiple number of the pulse separation should be determined to obtain the absolute longitudinal distance. This is achieved beforehand using the same spectral interferometry as the actual measurement with a slightly tuning the *f*_rep_ because when *f*_rep_ changes, the delay between the reference and probe pulses changes with an enhancement factor according to the number of pulses in the unbalanced path between the reference and probe arms in the interferometer^23^. This experiment demonstrates that the proposed method can measure long-range target shape using pulse-to-pulse interferometry without losing high accuracy.

## Discussion

First, the proposed method can provide high-resolution longitudinal shape measurements. In our experiments, we obtained an uncertainty of approximately 10 µm. The primary contributors to this uncertainty are attributed to be the spectral non-uniformity of the light source, the imperfect optical alignment of the spectral interferometer, the uncertainty in the motion of the mechanical stage used in the evaluation, and the analysis method. A mechanical stage is not required for the actual one-shot imaging; however, delay scanning is still required for calibration, which remains a source of error. This problem can be further solved via highly accurate delay scanning with OFC *f*_rep_ changes^23^. The non-uniform spectral shape negatively affected because the spectrum pattern was erroneously detected as a fringe. This problem can be overcome by flattening the spectrum of the light source. In addition, in measurements with the grating and camera, a high-resolution 2D spectrometer would help improve the image resolution. In this study, with further averaging in the beam transverse positions in the case of the step height measurement, such uncertainty contributions due to the optical spectrum and measurement system were smoothed out, and the distance measurement accuracy was subsequently improved to 1 µm. With further tailoring of the chirp degree and/or spectral resolution, even better accuracy could be realised.

The transverse spatial resolution in this demonstration was limited because we irradiated a collimated beam, i.e., a plane wave onto a target; this can be improved by use of imaging configuration in the optical setup with proper lenses. For example, using the proposed technique with a microscopic lenses, 3D shape of a microstructure can be observed. Moreover, imaging optical setup is essential when it is applied to diffusive objects in order to collect reflected probe light efficiently. In fact, 3D shape measurements of diffusive surfaces and microscopic structures based on the similar time-resolved imaging techniques using the imaging configuration have been reported in our previous studies^24–26^. The example of the optical setup for interferometric imaging of diffusive targets can be found in the setup for full-field optical coherence microscopy (FF-OCT)^27, 28^. In the FF-OCT techniques, the full-field interference images made by broadband light were obtained with focusing lenses such as microscopic objective^27, 28^. In the proposed method, in a similar way, broadband interference image can be detected by focusing the diffused probe light by focusing lenses.

Furthermore, using the developed spectroscopic imaging techniques^29–32^, such as with fibre bundle to convert a planar image to a linear configuration, it is possible to apply the proposed technique to arbitrary-aligned multi-points, i.e., 3D measurement by one shot.

We also considered the time resolution of the proposed method for capturing ultrafast phenomena. In our experiments, the chirp-free ultrashort pulse of 65-fs duration was used as the reference and the chirped femtosecond pulse is used as the probe, thus it can potentially be applied to ultrafast time-resolved imaging whose time resolution is determined by the reference pulse as in the case of the widely used pump-probe technique but captures non-repetitive phenomena^6^. Similar ultrafast time resolution can be also achieved by use of incoherent or noisy broadband nanosecond pulses^33^. Moreover, mode-locked laser generates train of pulses every 19.6 ns (51 MHz). In our experiments, we captured images using a camera (shutter speed: 30 fps), with each frame containing 1.7 million pulses. With improved detection system, the recording speed can be up to 51 Mfps.

Furthermore, the measurable range with one pulse is determined by the pulse width of the probe (5.7 ps), which extends 850 µm in space. In the experiments, the range was narrower owing to the shape of the light source spectrum and the wavelength range captured by the camera. However, using probe pulses with higher chirp, i.e., longer pulses, and a broad spectrum, the measurable range can be extended further. Moreover, as demonstrated in this study, step heights larger than one pulse width can be captured by pulse-to-pulse spectral interferometry. In addition, with the higher repetition rate of OFCs, the gap between the probe pulses can be reduced, and the measurable range can be further extended. For example, using a 1-ns chirp pulse train with a 1-GHz *f*_rep_, which is available with current technology, a target at an arbitrary position could be measured in one shot.

Finally, tailoring the OFC optical performances, such as repetition rate, spectral broadening and chirp ratio, allows the measurement performances of the depth resolution, measurement range, and time resolution to be tailored to the demand of the application. In this experiment, we stabilised *f*_ceo_ of OFC using widely-used 1f-2f interferometer to ensure the long-term stability for the precise evaluation of the newly developed method. However, simpler techniques to stabilise *f*_ceo_ by use of the pulse-to-pulse interference signal could also be used^34^.

## Conclusions

In this study, we proposed a new method to measure 3D shapes using one-shot imaging encoded in chirped OFCs. The proposed method involves measuring the longitudinal shape at each point on a target by detecting the interference fringe spectrum between the reference and chirped probe pulses reflected from the target using an image sensor. Subsequently, we measured the profile of the step height of two gauge blocks by capturing the spectral interferometric fringes without scanning, producing results that closely agree with the nominal value of 480 µm with an uncertainty of 12 µm. This result can be further improved by improving the optical, detection, and analysing systems, and the potential of the system has already been demonstrated by showing 1-µm uncertainty with further averaging in the transverse beam positions. Finally, we used pulse-to-pulse interferometry to demonstrate the simultaneous measurement of a large step height of approximately 3 m without increasing the uncertainty of 9.8 µm. Here, we demonstrated that the proposed method can be naturally extended to applications, such as moving targets, microscopic structure and objects with highly separated surfaces or high aspect ratios, which can significantly extend the dynamic range and versatility of the measurements. Through this study, we demonstrated an important building block for powerful one-shot 3D measurements with ultrafast time resolution, high precision and a large dynamic range by taking full advantage of OFCs.

## Methods

In this study, we analysed the interference fringe using two procedures called ‘spectrogram’ and ‘simplified analysis’.

To obtain the ‘spectrogram’ from the 2D plot (Fig. 3a), we selected 33 wavelength regions with widths of 3 nm and analysed each fringe pattern along the delay using a short-term Fourier transform. Consequently, a spectrogram as a function of the fringe frequency was obtained for each wavelength region. For each spectrogram, the delay position showing the MFF was obtained and plotted as a yellow point in Fig. 3a (labelled ‘Spectrogram’). As a result, from the 2D plot, we obtained the corresponding ‘wavelengths’ showing the MFFs for the 33 delay positions within the wavelength region of 1520–1620 nm. The uncertainties in the FFT analysis include errors owing to the finite width of the spectrum peaks obtained from the short-term Fourier transform. Optimizing the width of the Hamming window can reduce this effect. This analysis provides high accuracy because it is independent of the non-uniform shape of the light source’s optical spectrum. However, it requires consecutive delay changes for the FFT; therefore, we developed a second method as follows.

In the second analysis, called the ‘simplified analysis’, the fringe spectrum is analysed to obtain the MFF wavelength without changing the delay. Figure 3b shows one of the interference fringe spectra at a fixed delay position (indicated by the horizontal dashed line in Fig. 3a) after normalising according to the shape of the spectrum of the light source, removing a baseline and smoothing via a Savitzky–Golay filter, whose polynomial order and frame size were 2 and 45, respectively, to enhance the fringe structures^35^. In this example, our task was to obtain the wavelength region generating the MFF in the fringe spectrum, which is indicated by one of the vertical dashed lines near 1545 nm in Fig. 3b. Accordingly, the spectrum was differentiated by the wavelength and showed several zero-crossing points (Fig. 3c). It is evident that near the wavelength with the MFF, the interval between a pair of nearest zero-crossing points (Fig. 3d) is the largest. For simplicity, we fitted the plots with a Gaussian function and considered the peak to be the wavelength with the MFF. In this way, we obtained a series of wavelengths giving the MFF when the delay changed, which we plotted as a series of blue points in Fig. 3a (labelled ‘Simplified analysis’). Both analysis methods provided consistent results with similar levels of uncertainty.
